# Determinants of child anthropometric indicators in Ethiopia

**DOI:** 10.1186/s12889-018-5541-3

**Published:** 2018-05-15

**Authors:** Davod Ahmadi, Ekta Amarnani, Akankasha Sen, Narges Ebadi, Patrick Cortbaoui, Hugo Melgar-Quiñonez

**Affiliations:** 1 McGill Institute for Global Food Security, Macdonald Campus, 21111 Lakeshore Road, Ste-Anne-de-Bellevue, QC H9X 3V9 Canada; 2 Master student in School of Dietetics and Human Nutrition in McGill University, Montreal, Canada

**Keywords:** Child health, Child malnutrition, Developing countries, Food security, Nutrition status

## Abstract

**Background:**

Malnutrition is one of the major contributors to child mortality in Ethiopia. Currently established, child nutrition status is assessed by four anthropometric indicators. However, there are other factors affecting children’s anthropometric statuses. Thus, the main objective of this paper is to explore some of the determinants of child anthropometric indicators in Ethiopia.

**Methods:**

Data from GROW (the Growing Nutrition for Mothers and Children), a survey including 1261 mothers and 1261 children was carried out in Ethiopia in 2016. Based on the data gathered, the goal of GROW is to improve the nutritional status of women of reproductive age (15–49), as well as boys and girls under 5 years of age in Ethiopia. In order to investigate the association between different factors and child anthropometric indicators, this study employs various statistical methods, such as ANOVA, T-test, and linear regressions.

**Results:**

Child’s sex (confidence intervals for (wasting = − 0.782, − 0.151; stunting = − 0.936,-0.243) (underweight = − 0.530, − 0.008), child’s age (confidence intervals for (wasting = − 0.020, 0.007; stunting = − 0.042,-0.011) (underweight = − 0.025, − 0.002), maternal MUAC (confidence intervals for (wasting = 0.189, 0.985; BMI-for-age = 0.077, 0.895), maternal education (stunting = 0.095, 0.897; underweight = 0.120, 0.729), and open defecation (stunting = 0.055, 0.332; underweight = 0.042, 0.257) were found to be significantly associated with anthropometric indicators. Contrary to some findings, maternal dietary diversity does not present significance in aforementioned child anthropometric indicators.

**Conclusion:**

Depending on the choice of children anthropometric indicator, different conclusions were drawn demonstrating the association between each factor to child nutritional status. Results showed child’s sex, age, region, open defecation, and maternal MUAC significantly increases the risk of child anthropometric indicators. Highlighting the factors influencing child undernutrition will help inform future policies and programs designed to approach this major problem in Ethiopia.

## Background

Malnutrition in children results in a higher disease burden and increased levels of mortality [1]. Recent evidence has shown that malnutrition is the major risk factor for children under–five years of age [2–4]. Child malnutrition is characterized with four indicators [5]: stunting (low height for age), wasting (low weight for height), underweight (low weight for age), and BMI-for-age (height and weight for age). Stunting is reflective of the cumulative effects of poor nutrition or infections before or since birth; on the other hand, wasting is a measure of acute malnutrition, and is usually due to insufficient food intake or an acute onset of infectious diseases. Underweight and low BMI-for-age can reflect wasting, and are associated with an increased risk of mortality [6]. As the most prevalent form of child malnutrition, stunting affects millions of children globally [7]. Ultimately, child malnutrition impacts the growth, mortality, cognitive ability, and psychosocial development of the child [8].

As of the moment, approximately 25 % of Ethiopian sub-regions (Woredas) are officially in nutritional crises. Specifically, children and women (pregnant and lactating) are in urgent needs for supplementary feeding and treatments of severe acute malnutrition [9, 10].

In Ethiopia, progress made in the reduction of child malnutrition in the last two decades were hindered at multiple levels, such as environmental (drought), political (war) and economic factors (low agricultural production, low GDP) [11, 12]. Traditionally, child undernutrition was viewed as a consequence of food shortages [13].

As the primary demographic variables, age and sex are important determinants of stunting. From chronic malnutrition studies, analysis at different ages reveals how nutritional status changes during the first three years of life [14]. Maintaining basic child dietary diversity by consuming essential nutrients and higher energy contributes directly to growth and cognitive development [15]. Maternal characteristics such as dietary diversity, [16] education, [17] Mid-Upper-Arm Circumstance (MUAC), [18] and adequacy of breastfeeding or complementary feeding practices [19–21] are also strongly linked to child anthropometric statuses.

When considering other factors influencing child growth, poor water, sanitation, and hygiene practices also play the roles of increasing health risks such as consequential diarrhea from water-borne infections [14]. According to *Fenn* et al. [22], improvement in sanitation is associated with reduction of stunting. When compared, children with access to toilets demonstrated lower rate of stunting to those who defecate in the open [23].

As previously stated, existing studies evaluate the effects of risk factors on either stunting, wasting, undernutrition, or BMI-for-age. Yet limited research has been shown to understand how these determinants affect all four indicators when examined together. Therefore, the purpose of this study is to assess the factors associated with the aforementioned anthropometric indications in Ethiopian children, potentially serving as base line studies for future child nutrition driven interventions.

## Methods

### Data sources

The data for this study was gathered from a baseline survey GROW (the Growing Nutrition for Mothers and Children) in 2016. The goal of GROW is to improve the nutritional status of women of reproductive age, as well as boys and girls under 5 years of age in Ethiopia.

### Sample size

The sample was chosen by randomly selecting 14 sub-regions (Woredas) within each of the three Ethiopian regions (Afar, West Hararghe, and East Hararghe). Within each of the 14 sub-regions, 39 suitable villages (Kebeles) were randomly selected, of which the survey was administered to a random sample of households with children under the age of 5.

The sampling frame for this survey was constructed using Kebele level population data obtained from government census, district and local office sources within the 12 intervention Woredas. Accordingly, 39 Kebeles were selected from 164 intervention Kebeles using probability proportional to size (PPS) technique with a total population of 276,804 as provided in the sampling frame. The calculated sample size (1310) was allocated across the 39 selected Kebeles using PPS technique based on the population size of Kebeles.

A community-based cross-sectional observational study design was employed. For children, the sample size was calculated using prevalence of key infant and young child feeding (IYCF) based on exclusive breastfeeding practices, and a target percentage point change was expected to take place at the end of the proposed intervention. For the IYCF indicators, exclusive breastfeeding was used as the key indicator for sample size determination.

In addition to mothers’ and fathers’ information, household characteristics, children dietary diversity, and anthropometric information were recorded. The questionnaire was validated by local experts using focus groups to ensure the translation was true to the original intent of each of the survey items. In summation, a sample of 2522 women and children was selected for this study.

### Outcome variables

Children were classified as stunted, wasted, underweight, or low BMI-for age (dependent variables) based on their Z-scores for weight-for-height (WHZ), height-for-age (HAZ), weight-for-age (WAZ), and body-mass-index-for-age (BMIZ). Children were considered stunted, wasted, or underweight if their Z-scores were less than − 2 SD below the median values in reference to the United Nations World Health Organization [24].

### Exposure variables

Factors that have been associated with child malnutrition in the literature were included as exposure variables in the study: maternal education, working outside, food security status, maternal dietary diversity, and maternal mid upper arm circumference (MUAC). Children’s sex, age groups (i.e., 6–11; 12–23; 24–59)^1^, experience of diarrhea in the last two weeks (i.e., yes, no), access to sanitation facilities, and defecate openly^2^ were included in the analysis.

### Maternal characteristics

Maternal age, as a scale variable, was recoded into four age groups: 15–24, 25–29, 30–39, and 40–49. Due to the large number of mothers who had never obtained formal education, it was recoded into two answer groups as “no education” and “have any education”. Women’s working status was evaluated using the question, “Do you work outside?” (i.e., “yes” and “no”). Maternal marital status was categorized from “married monogamous”, “married polygamous”, “divorced”, “widowed”, “single/never married”, “cohabitating with monogamous partner”, “cohabitating with polygamous partner”, into “married/living with partner” and “single/divorced”, due to the small number of mothers in the other marital status groups.

Food security status was measured using the *Food Insecurity Experience Scale* (FIES), a tool produced and validated worldwide by the Food and Agriculture Organization of the United Nations. The FIES is composed of eight psycho-metric questions focusing on measuring the access aspect of food security. Responses to the eight dichotomous questions were summed (1 = Yes and 0 = No), and produced into a new variable based on the summation, ranging from 0 to 8. Those who answered negatively to all eight questions (score of 0) were classified as food secure. Those who answered affirmatively to one to three questions, four to six questions, or seven to eight questions were classified as “mildly food insecure,” “moderately food insecure,” or “severely food insecure” respectively [25].

To create maternal dietary diversity scores, questions related to the consumption of starchy staples, dark green leafy vegetables, other vitamin A rich fruits and vegetables, other fruits and vegetables, organ meats, meat and fish, eggs, legumes, nuts and seeds, milk and milk products were asked. The scores were summed, and a new score based on the summation was produced and categorized into two groups: “0–3”, “4–9” [24].

### Children characteristics

Children’s ages were grouped into three categories: 6–11 months (complementary feeding), 12–23 months (transition from complementary feeding to regular), and 24–59 months (regular household diet).^3^ By using questions asking about children dietary diversity, the following seven food groups were summed: grains, roots and tubers, legumes and nuts, dairy products (milk, yogurt, cheese), flesh foods (meat, fish, poultry and liver/organ meats), eggs, vitamin-A rich fruits and vegetables, and other fruits and vegetables. Categories based on the summation were created- a score of zero to three indicating low diet diversity, and four to seven indicating high diet diversity [26].

### Household characteristics

Household (HH)-related characteristics such as, head of HH, number of children under 5, HH size, HH electricity condition, and sanitation facilities were assessed in this study.

### Data analysis

Descriptive statistics were used to examine the frequency of dependent^4^ and independent variables. A one-way ANOVA and t-test were used to compare the mean and SD of independent variables for each of the anthropometric indicators; differences in means were evaluated using f-tests and t-tests. Lastly, four linear regression analyses were carried out to explore which factors are the major determinants of children’s anthropometric indicators.

## Results

Socio-demographic characteristics of households, mothers and children (under 5) in study population are presented in Table 1. Seventy percent of mothers were between 25 and 39 years of age. Notably, majority of mothers were either married or living with a partner (96%), uneducated (21%), and working outside the home environment (36%). Results for food insecurity status showed that 31 % of mothers were food secure (31%), and around one quarter (24%) were malnourished, according to their MUAC. The majority of children had low dietary diversity (80.1%). In terms of child anthropometry, around two-thirds of children were stunted (67.4%), one-third underweight (31.6%), one-fifth wasted (20.7%), and one-fifth had low BMI-for-age (20.4%).

**Table 1:** Maternal and household characteristics (*n* = 1261)

			N (%)
Maternal characteristics	Age	15–24	318 (25.2)
		25–29	477 (37.8)
		30–39	413 (32.8)
		40–49	53 (4.2)
	Education	Having education	261 (20.8)
	Marital	Married/living with partner	1208 (95.8)
	Work outside	Yes	454 (36.0)
	FIES	Food security	302 (30.8)
		Mild food insecurity	200 (20.4)
		Moderate food insecurity	208 (21.2)
		Severe food insecurity	270 (27.6)
	Maternal MUAC (Centimeter)	13–23 (Centimeter)	306 (24.3)
Household characteristics	Region	Afar	286 (22.7)
		East Hararghe	502 (39.8)
		West Hararghe	473 (37.5)
	Head of HH	Male	1120 (88.8)
	Number of children under 5	1	368 (29.2)
		2	620 (49.2)
		3	209 (16.6)
		4–10	64 (5.1)
	Number of people in HH	1–2	12 (1.0)
		3	158 (12.5)
		4	164 (13.0)
		5	254 (20.1)
		6–17	673 (53.4)
	HH Has electricity	Yes	571 (45.8)
	Sanitation facilities	Improved sanitation facilities	259 (20.5)
		Open defecation	442 (35.1)
	Sex	Male	713 (56.5)
		Female	548 (43.5)
	Age	6–23	265 (21.0)
		24–59	265 (210)
	Diarrhea in last two weeks	Yes	192 (15.2)
	Children dietary diversity	0–3	766 (80.1)
		4–7	190 (19.9)
	Children Anthropometric	Wasting -2SD	135 (15.4)
		Wasting -3SD	52 (5.3)
		Underweight -2SD	205 (21.9)
		Underweight -3SD	91 (9.7)
		Stunting -2SD	375 (41.7)
		Stunting -3SD	231 (25.7)
		Bmi for age -2SD	123 (14.2)
		Bmi for age -3SD	54 (6.2)

*Reference*: Self-calculation of micro-data from Survey of Ethiopia 2016 (GROW)

Results of t-test and ANOVA analyses are presented in Table 2. T-test analysis showed that each anthropometric indicator, except BMI-for-age, was associated statistically with child’s gender. Importantly, boys had relative poor status when compared to their female counterparts. Significant differences were found between age groups and anthropometric indicators, except wasting. ANOVA analysis indicated stunting and underweighting were more prevalent in children between age 24 and 59. There was furthermore a significant difference between BMI-for age and open defecation. Regional differences were found for children’s anthropometric indicators; children living in West Hararghe demonstrated the best status for all four indicators amongst other regions.

**Table 2:** One-way Anova- and T-test analyses between anthropometric indicators and independent variables (*n* = 1005)

			Wasting (weight for height)	stunting (height for age)	underweight (weight for age)	BMI for age
Sex of children	Female	Mean	0.1484	−1.0629	−0.5610	0.2111
		SD	1.8437	2.2316	1.6612	1.8923
	Male	Mean	−0.1650	−1.5870	− 0.9580	− 0.0181
		SD	−0.2325	2.2905	1.7326	2.1102
		T-Test	**	**	***	n.s
Children age groups	6–11	Mean	−0.2064	− 0.5442	− 0.7360	− 0.2463
		SD	2.2475	2.5809	1.9379	2.2847
	12–23	Mean	0.0963	−1.6473	−0.6868	0.2940
		SD	1.9181	2.2235	1.7390	1.9574
	24–59	Mean	−0.2099	−1.5841	−1.0242	−0.0072
		SD	1.6772	1.9117	1.4096	1.8461
		Anova F-Test	n.s	***	*	**
Children Diarrhea in the last two weeks	No	Mean	−0.0518	−1.4115	−0.7895	0.0758
		SD	1.9372	2.3108	1.6909	2.0274
	Yes	Mean	−0.1919	−1.1421	− 0.8469	0.0109
		SD	1.9416	2.1070	1.8231	1.9541
		T-Test	n.s	n.s	n.s	n.s
Children dietary diversity	0–3	Mean	−0.0249	−1.3540	− 0.7196	0.1132
		SD	1.9155	2.2560	1.6952	2.0104
	4–7	Mean	−0.1292	−1.2584	− 0.8714	− 0.0069
		SD	1.9861	2.3718	1.6545	1.9824
		T-Test	n.s	n.s	n.s	n.s
Open defecation	No	Mean	−0.1344	−1.6229	− 0.7763	0.2890
		SD	1.9714	2.1786	1.7479	2.0103
	Yes	Mean	−0.1408	−1.3276	−0.7327	0.0677
		SD	2.0227	2.2673	1.7080	2.1490
		T-Test	n.s	n.s	n.s	**
Region	Afar	Mean	−0.7815	−1.0404	−1.1039	−0.7053
		SD	1.8165	2.3252	1.5704	1.9519
	East Hararghe	Mean	0.1154	−2.0501	−1.0630	0.3189
		SD	1.9477	2.0433	1.6684	1.9991
	West Hararghe	Mean	0.2112	−0.8652	−0.3076	0.3257
		SD	1.9145	2.3130	1.7391	1.97044
		Anova F-Test	***	***	***	***
Maternal education	No education	Mean	−0.1355	−1.4260	− 0.8693	0.0116
		SD	1.9710	2.2208	1.7637	2.0353
	Have education	Mean	0.2331	−1.1238	−0.4727	0.3394
		SD	1.8248	2.4981	1.4650	1.9673
		T-Test	*	n.s	**	*
Maternal Muac (centimeter)	13–22	Mean	−0.5964	−1.4673	−1.3404	− 0.4337
		SD	1.8520	2.2942	1.6320	1.9981
	23–34	Mean	0.1342	−1.3337	−0.6002	0.2668
		SD	1.9432	2.2752	1.7006	2.0021
		T-Test	***	n.s	***	***
Maternal dietary diversity	0–3	Mean	−0.0711	−1.3899	−0.7905	0.0797
		SD	1.9593	2.2551	1.7291	2.0500
	4–9	Mean	−0.0374	−1.3163	−0.7903	0.0762
		SD	1.9159	2.3378	1.6797	1.9623
		T-Test	n.s	n.s	n.s	n.s

Chi-square significance: * = *p* < 0.05; ** = *p* < 0.01; *** = *p* < 0.001; n. s. not significant at the threshold of 0.05; *n.s* Non significant

*Source:* Self-calculation of micro-data from Survey of Ethiopia 2016 (GROW)

An inverse relationship between maternal education and children’s anthropometric indicators status (except stunting) was observed in this study. Evidently, educated mothers appeared to have children with low levels of wasting, undernutrition and BMI-for-age when compared to mothers with no education. Additionally, significant association were found between maternal MUAC, child wasting, child underweight, and child low BMI-for-age.

Table 3 displays linear regression results for the association between child anthropometric indicators statuses and some predicting factors. Results showed that sex of children (− 0.114; *P* = 0.004), maternal MUAC (0.113; *P* = 0.004), and region of living (0.141; *P* = 0.000) remained significantly associated with prevalence of wasting. Further, sex (− 0.126; *P* = 0.001), age (− 0.128; *P* = 0.001), diarrhea in the last two weeks (0.081; *P* = 0.034), open defecation (0.103; *P* = 0.006), and maternal education (0.092; *P* = 0.015) contributed to the level of stunting. Results for BMI-for-age indicated that sex (− 0.074; *P* = 0.043), age (− 0.084; *P* = 0.021), open defecation (0.100; *P* = 0.006), maternal education (0.100; *P* = 0.006), and MUAC (0.136; *P* = 0.000) remained significant. Also, maternal MUAC (0.091; *P* = 0.020) and region of living (0.150; *P* = 0.000) were significantly associated with BMI-for-age. It is conclusive that girls had better anthropometric statuses comparing to their male counterparts. Also, open defecation was negatively associated with children’s anthropometric status, indicating that open defecation directly contributes to degrading statuses. Children living in the Afar region had the poorest nutritional status, relatively in this study. Furthermore, mothers with better MUAC and higher education level had children with better nutritional statuses.

**Table 3:** Linear regression analyses between anthropometric indicators and independent variables (*n* = 1005)

		Wasting^a^ (weight for height)		stunting^b^ (height for age)		underweight^c^ (weight for age)		BMI^d^ for age	
		Beta	L-H bound	Beta	L-H bound	Beta	L-H bound	Beta	L-H bound
Independent	Sex of children	−0.114	−0.782, −0.151	−0.126	−0.936,-0.243	− 0.074	−0.530,-0.008	− 0.071	−0.627,0.022
variables	Age of children	−0.036	−0.020,0.007	− 0.128	−0.042,-0.011	− 0.084	−0.025,-0.002	− 0.041	−0.022,0.007
	Diarrhea in last two weeks	−0.034	−0.629,0.241	0.081	0.038,0.975	− 0.029	−0.501,0.217	− 0.019	−0.568,0.345
	Open defecation	0.007	−0.116,0.138	0.103	0.055,0.332	0.100	0.042,0.257	−0.006	− 0.143,0.121
	Region	0.141	0.548-,1.840	−0.064	−1.349,0.099	0.061	−0.087,1.037	0.150	0.647,1.989
	Maternal education	0.045	−0.150,0.585	0.092	0.095,0.897	0.100	0.120,0.729	0.054	−0.1100.641
	Maternal MUAC	0.113	0.189,0.985	0.057	−0.106,0.794	0.136	0.305,0.983	0.091	0.077,0.895
	Women’s dietary diversity	−0.040	−0.516,0.166	0.032	− 0.216,0.534	−0.031	− 0.402,0.161	−0.051	− 0.578,0.122

Children’s dietary diversity was removed from the model due to collinearity with women’s dietary diversity

^a^ Model summary (*R* = 0.240; R^2^ = 0.057; adjusted R square = 0.046; Std. Error of the estimate = 1.973; Durbin Watson = 1.719)

^b^Model summary (*R* = 0.247; R^2^ = 0.061; adjusted R square = 0.050; Std. Error of the estimate = 2.242; Durbin Watson = 1.681)

^c^Model summary (*R* = 0.234; R^2^ = 0.055; adjusted R square = 0.044; Std. Error of the estimate = 1.745; Durbin Watson = 1.740)

^d^Model summary (*R* = 0.217; R^2^ = 0.047; adjusted R square = 0.035; Std. Error of the estimate = 2.050; Durbin Watson = 1.675)

*Source:* Self-calculation of micro-data from Survey of Ethiopia 2016 (GROW)

## Discussion

More than one-quarter of Ethiopian sub-regions are in nutritional crises. So, the purpose of this study was to assess the factors associated with the anthropometric indications in Ethiopian children.

Boys had a higher prevalence of wasting, stunting, and underweight when compared to girls; no significant differences in the prevalence of low BMI-for-age were seen between sexes. These findings are corroborated by literature, demonstrating that boys are at higher risk of preterm birth [27] and exposure to environmental stress (repeated infections, and increased exposure to toxins and air pollutants) [28]. These factors potentially influence height and weight, and seems to explain the difference in anthropometric indicators between boys and girls.

While no differences were found in the prevalence of wasting between children age groups, significant differences were seen for the prevalence of stunting, underweight, and low BMI-for-age. Children of 12–59 months showed the highest risk of stunting amongst other groups. A 2016 study conducted by Gebreyesus and colleagues [29] found similar trends, with older children demonstrating a higher risk of stunting than younger children. Conversely, this study found the youngest age group had the highest risk of low BMI-for-age. Limited studies explaining this trend are available, thus this study emphasizes the importance of findings explaining the complex nature of stunting and low BMI-for-age.

In terms of children diarrhea, neither children with diarrhea nor those without showed statistical association to the prevalence of stunting, wasting, underweight, and BMI-for-age. A study in rural Rwanda found similar results, the risks of stunting and wasting in children with diarrhea were not significantly different than their counterparts [30]. While diarrhea is a contributing factor to undernutrition, it alone does not fully explain stunting, wasting, undernutrition, and BMI-for-age, particularly when the reference period is relatively short (14 days) [31].

No significant differences in the risks of stunting, wasting, undernutrition, and BMI-for-age were seen amongst children with low and high dietary diversity scores. A 2013 study conducted by Ali and colleagues in Ethiopia, Vietnam, and Bangladesh [32] also found no representing association between child dietary diversity and anthropometric indicators; meaning that child dietary diversity is unlikely to have a large enough influence on nutrition to impact anthropometric indicators.

Children who defecated in the open had a significantly higher risk of having low BMI-for-age, but no significant differences when considering risks of wasting, stunting, or undernutrition. While this research only found significant associations between BMI-for-age and open defecation, it is complimented by previous researches in developing countries which found defecation in the open posing as threats for child linear growth [33]. The mechanisms behind this were attributed to diminished appetite, impaired nutrient absorption, and increased nutrient losses due to diarrhea [34]. Regional differences in the risks of stunting, wasting, undernutrition, and low BMI-for-age were presented in the previous section; children living in West Hararghe had the lowest risks of all four indicators when compared to those in Afar and East Hararghe. The poorest nutritional status of the children living in the Afar region may be reflective of the political disregard and poor living standards in the region [35].

Children with mothers who had no formal education had a higher risk of wasting, underweight, and low BMI-for-age, but not stunting. While some studies have found maternal education to be associated with all anthropometric indicators [36], others have found maternal education to be associated with only some anthropometric indicators. A study conducted in Ghana demonstrated the association between maternal knowledge and the child’s long-term wellbeing (height-for-age), but not with underweight [37]. The relationship between maternal education and anthropometric indicators may affect a child differently long-term and during an acute illness, explaining the fluctuations seemed in the child’s growth indicators.

Similar to education, maternal MUAC was significantly associated with child wasting, underweight, and low BMI-for-age, but not stunting. A recent study demonstrated that beyond maternal weight as a predictor of child nutrition status, the mother’s vitamin and mineral status also play a role. The study found that mothers lacking certain nutrients in their breastmilk were likely to have children who were stunted. Conversely, mothers with no deficiencies of nutrients in their breastmilk did not have children that were significantly more stunted [38]. Since MUAC is an indicator of malnutrition that does not take into account specific nutrient deficiency, this may explain why maternal MUAC does not have the same effect on all the child anthropometric indicators. Maternal dietary diversity, much like the child’s dietary diversity, was not found to be significantly associated with any of the child’s anthropometric indicators.

In this study, analyses were carried out to explore the determinants of malnutrition that are profoundly related to child mortality in Ethiopia. The superior nutritional statues of girls under the same household conditions warrant further investigations. As confirmed in this study, stunting, wasting, undernutrition, and low BMI-for-age remains alarming in Ethiopia. Highlighting the factors influencing child undernutrition will help inform future policies and programs designed to approach this major problem in Ethiopia.

### Limitations

Due to the cross-sectional nature of this study, the direction of associations cannot be firmly established. Additionally, constrained by the short reference period for diarrhea, the long-term association between diarrhea and child nutrition may not be representative. Lastly, it is suggested that future research is to focus on the influence of interactions between independent variables on the child anthropometric indicators.

## Conclusion

This study found that maternal and children’s characteristics influence the anthropometric indicators. Results showed that child’s sex, age, region, open defecation, and maternal MUAC significantly increases the risk of wasting, stunting, undernutrition, and/or BMI-for-age. Importantly, maternal dietary diversity was not significant amongst other determinants of children’s anthropometric statues. Given the numerous child anthropometric indicators available, this study highlights the influence of controlling variables such as, sex, age, and region used for nutritional status in our study. Thus, caution must be used when interpreting research using single indicators to draw conclusions on child nutrition status.

**Table 4:** Test of data normality

	Kolmogorov-Smirnov			Shaprio-Wilk		
	Stataitics	df	sig	Stataitics	df	sig
Wasting	0.020	917	0.200	0.996	917	0.009
Stunting	0.033	917	0.021	0.987	917	0.000
Underweight	0.026	917	0.143	0.998	917	0.213
BMI	0.020	917	0.200	0.996	917	0.023

*Source:* Self-calculation of micro-data from Survey of Ethiopia 2016 (GROW)

## Footnotes

1 In the regression analyses, age was used in a scale level.

2 This question was used: Do any of your household members defecate in the bush/field? With yes and no answers.

3 Although this indicator is recommended for children under 24 months, children up to 59 were added in the analyses.

4 Normality of data was analyzed using SPSS (V.24). Table of data normality presented in Table 4.
